# Thyroid diseases in children and adolescents requiring surgical treatment—indications, techniques, results, and complications based on 10 years of the single center’s own experience

**DOI:** 10.3389/fendo.2023.1301191

**Published:** 2024-01-12

**Authors:** Patrycja Sosnowska-Sienkiewicz, Dajana Danielewicz, Danuta Januszkiewicz-Lewandowska, Paulina Rusak, Iwona Anderko, Maciej Rzepecki, Marek Niedziela, Jerzy Harasymczuk, Przemysław Mańkowski

**Affiliations:** ^1^ Department of Pediatric Surgery, Traumatology and Urology, Poznan University of Medical Sciences, Poznań, Poland; ^2^ Department of Pediatric Oncology, Hematology and Transplantology, Poznan University of Medical Sciences, Poznań, Poland; ^3^ Student Research Group of Pediatric Surgery, Poznan University of Medical Sciences, Poznań, Poland; ^4^ Department of Pediatric Endocrinology and Rheumatology, Poznan University of Medical Sciences, Poznań, Poland

**Keywords:** child, complications, outcomes, thyroid gland, thyroidectomy

## Abstract

**Introduction:**

Although thyroid abnormalities are observed less frequently in children than in adults, the increased incidence of thyroid cancer makes it mandatory for all pediatric surgeons to be knowledgeable about the disorders of this gland. Thyroid abnormalities can be associated with hyperthyroidism or hypothyroidism and euthyroidism and/or symmetric or asymmetric enlargement of the gland.

**Aim:**

The present study was undertaken to retrospectively analyze the indications, surgical techniques used, results obtained, and complications found in the surgical treatment of thyroid diseases in children and adolescents in a surgical center for the macro-region of western Poland.

**Methods:**

The data of 148 patients undergoing total or partial thyroidectomy between 2013 and 2022 were analyzed from the medical records of the Department of Pediatric Surgery, Traumatology, and Urology of the Medical University of Poznan, Poland.

**Results:**

A total of 95 children underwent subtotal thyroidectomy and 64 underwent total thyroidectomy, of which the procedure was widened to include prophylactic removal of neck lymph nodes in 45 patients. There were 113 girls (76%) in the analyzed group, and the average age of the patients at the time of surgical treatment was 15 years. The average time from the diagnosis of thyroid disease to surgery was 4 months, ranging from 2 weeks to 3 years. Of the 64 patients undergoing total thyroid resection, 35 (54.69%) were diagnosed with thyroid cancer.

**Conclusions:**

Collaboration within a multidisciplinary team ensures optimal surgical outcomes in children and adolescents with thyroid disease. With extreme caution, thyroid removal is a safe procedure with few complications, but the experience of the surgeon performing thyroid surgery in children remains crucial. Despite the absence of such a diagnosis in the first fine-needle aspiration biopsy, the high percentage of thyroid carcinomas in the analyzed group may be because the initial biopsy was performed in a less experienced center, also in terms of histopathological laboratory. Hence, we point out the necessity of performing a repeat fine-needle aspiration biopsy (according to the Bethesda classification) in a more experienced center before the final decision of thyroidectomy.

## Introduction

Thyroid diseases are an increasing problem in children, adolescents, and adults, affecting a growing group of patients. Although thyroid diseases are relatively infrequently described in children, compared to adult patients, the increased incidence in the pediatric group should contribute to increased awareness among pediatric surgeons, resulting in proper qualification for surgical treatment and improved prognosis (1–3). The basis for the diagnosis of thyroid disease is a physical examination, including consideration of family history of the disease. Thyroid hormone tests—TSH, FT3, FT4, anti-TSHR, anti-TPO, anti-TG antibodies, thyroglobulin, and calcitonin—are also important (1). The imaging studies include thyroid ultrasound and thyroid scintigraphy (2, 4). Fine-needle aspiration biopsy (FNAB), performed under ultrasound guidance, plays an important role (2–5). **Table 1** shows the Bethesda system with diagnostic thyroid cytopathology and clinical recommendations.

**Table 1:** The Bethesda system with diagnostic thyroid cytopathology and clinical recommendations.

Diagnostic Category	Risk of Malignancy (%)	Usual Management
Nondiagnostic or unsatisfactory	5–10	Repeat FNA with ultrasound guidance
Benign	0–3	Clinical follow-up
Atypia of undetermined significance or follicular lesion of undetermined significance	6–18	Repeat FNA, molecular testing, or lobectomy
Follicular neoplasm or suspicious for a follicular neoplasm	10–40	Molecular testing, lobectomy
Suspicious for malignancy	45–60	Near total thyroidectomy or lobectomy
Malignant	94–96	Near total thyroidectomy or lobectomy

Surgical treatment and the extent of resection of the thyroid gland depend on the diagnosis, accompanying clinical manifestations, and the involved area(s) of the thyroid gland. Owing to symptomatic compression of surrounding structures such as the trachea or esophagus, a colloid, nodular, or multinodular thyroid goiter may require surgery to reduce symptoms and improve the patient’s quality of life (1). In addition, surgery may also be recommended for nodular goiter when imaging studies such as ultrasonography or the result of a fine-needle biopsy suggest the presence of malignant lesions (1, 2). Thyroidectomy may be necessary in autoimmune and functional thyroid diseases involving the entire thyroid gland. These include hyperthyroidism, unresponsive to treatment, or advanced autoimmune disorders in which the thyroid gland is significantly damaged and nodularly altered (1, 3). The thyroid gland can also become hyperactive due to the presence of autonomous adenomas. Thyrotoxicosis can also result from the use of amiodarone, an iodine-containing drug (6).

Surgical treatment is the treatment of choice for patients with precancerous lesions and confirmed thyroid cancer (2, 4, 7, 8). In children and adolescents, papillary and follicular thyroid carcinomas account for approximately 90% of all malignant thyroid tumors (4). Thyroid cancer staging is performed using the TNM (Tumor-Node-Metastasis) classification according to the 8th edition of the UICC (Union for International Cancer Control). There are two stages of papillary and follicular thyroid cancer (stage I includes each T, each N with M0, and stage II includes every T, every N of M1) (2). Tumor grade and histologic type are considered in determining the extent of surgery. However, the method of choice is total thyroidectomy, often in addition to the removal of regional neck lymph nodes (2). Prophylactic total thyroidectomy is performed when there is an increased risk of genetically based cancer, such as that caused by germline mutations of the RET protooncogene (5).

Long-term complications of total and partial thyroidectomy are not common, and perioperative mortality is very low (4, 9–12). The most often observed postoperative complication is hypoparathyroidism with associated hypocalcemia (8–11). A higher incidence of hypoparathyroidism has been described after total thyroidectomy with simultaneous removal of regional lymph nodes (9, 10). Other complications include bleeding, wound infection, and injury of the posterior laryngeal nerves and the vocal cords (9).

## Aim of the study

The present study was undertaken to retrospectively analyze the indications for thyroidectomy, surgical techniques used, results obtained, and complications observed after surgical treatment of thyroid diseases in children and adolescents in a surgical center for the macro-region of western Poland.

## Materials and methods

Data were collected from the medical records of patients treated in the Department of Pediatric Surgery, Traumatology, and Urology at the Poznan University of Medical Sciences from 2013 to 2022. A total of 148 patients underwent surgical treatment—total or subtotal thyroidectomy. A total of 11 children required reoperation, including 3 cases due to recurrence of disease, and in 8 patients, the initial histopathological result indicated the need for extended resection. Based on laboratory tests performed, patients were defined as having hyperthyroidism, euthyroidism, or pharmacologically controlled hypothyroidism. According to published recommendations, patients were qualified based on ultrasound and laboratory tests for thin-needle biopsy, subtotal, or total thyroidectomy with or without lymph node excision (13–18). Analysis included age, gender, comorbidities, laboratory tests, indications for surgery, preoperative period (need for pharmacotherapy before surgery and other treatments from history), type of surgery (primary surgery, reoperation, duration of surgery, need for blood transfusion, occurrence of complication, and type of complication), postoperative period (complications, need for additional therapy, duration of hospitalization, reported pain on a scale of 1–10, pain medications used resulting in relief of pain, and blood transfusion during hospitalization after surgery), and histopathological diagnosis.

Each patient underwent surgical evaluation 7–10 days and 2 months after surgery. Patients remained under constant follow-up by an endocrinologist or an oncologist and had regular laboratory tests and ultrasound examinations until they were 18 years old.

### Surgery technique

The types of thyroid surgery used included subtotal thyroidectomy, which was the removal of one lobe of the thyroid gland, including the isthmus, and total thyroidectomy, which was the removal of both lobes of the thyroid gland, including the isthmus.

### Subtotal thyroidectomy

At the base of the neck, approximately 2 cm above the cervical notch of the sternum, an incision of approximately 4 cm is made. The length of the incision depends on the size of the thyroid gland to be operated on. Such an incision provides adequate access to the organ, vascular course, and adjacent structures necessary for visualization. The next step is to release—to detach the thyroid gland from the surrounding tissues—muscles and other soft tissues. The vessels (the superior and inferior thyroid arteries) should be carefully worked out. They could be secured by coagulating or puncturing, depending on the diameter of the vessel and the operator’s beliefs. Critical to the operation is the correct identification of the recurrent laryngeal nerve. According to the latest standards, the recurrent laryngeal nerve and the superior laryngeal nerve must be identified by neuromonitoring. With special care, the first lobe of the gland is dissected, reaching the expected location of the recurrent laryngeal nerve. The recurrent laryngeal nerves lie on both sides of the trachea; they then reach the posterior surface of the thyroid gland in the groove between the trachea and the esophagus. The recurrent laryngeal nerve is dissected, remembering not to coagulate near it and to stop the bleeding by ligating or puncturing the bleeding vessel. During the operation, we identified the parathyroid glands. After excision of the gland lobe, the isthmus of the thyroid gland should be exposed and dissected. The procedure is completed by checking for hemostasis. Tranexamic acid is used in infiltrative form after removal of the thyroid lobe, including the isthmus. Layered tissue reconstruction completes the operation.

### Total thyroidectomy

Depending on the size of the thyroid gland, the surgical incision must be extended by approximately 1–2 cm. The steps described above are repeated on the side of the second operated lobe, constantly with due care, remembering intraoperative neuromonitoring. Drains are currently used sporadically.

### Statistical analysis

Calculations were made using Statistica 13 by TIBCO and PQStat by PQStat Software. The significance level was α = 0.05. The result was considered statistically significant when *p* < α. The normality of the distribution of variables was tested with the Shapiro–Wilk test. The Mann–Whitney test was calculated to compare the variables between the 2 groups. Spearman's rank correlation coefficient (*Rs*) was calculated to examine the relationship between the variables. The χ^2^ test of independence, Fisher’s exact test, or Fisher–Freeman–Halton test was calculated to investigate the relationship between categorical variables. The odds ratio was calculated with a 95% confidence interval if there was a relationship. Cohen’s kappa concordance coefficient was calculated to assess the concordance of the initial and final diagnosis.

The study was approved by the Bioethics Committee of the Poznan University of Medical Sciences (Resolution no. KB-752/23, 22 September 2023).

## Results

**Figure 1:**
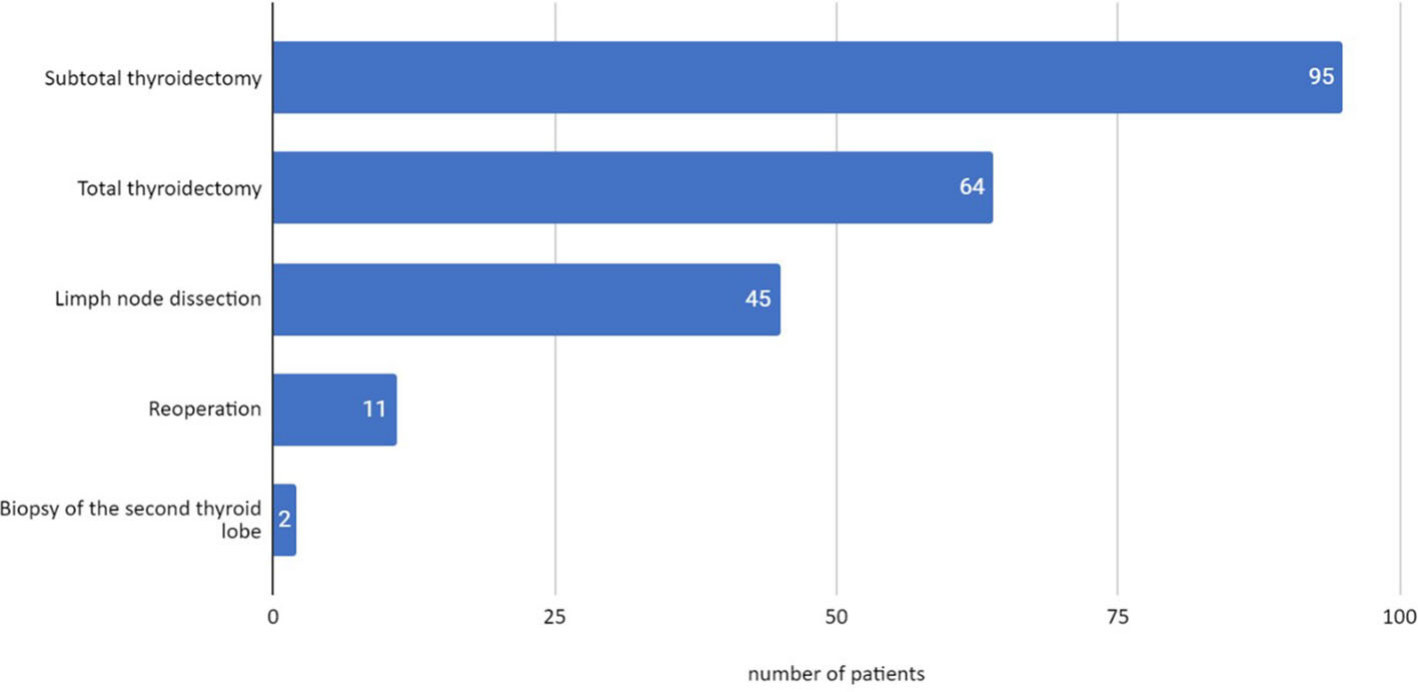
Types of surgical procedures performed in the analyzed patients.

In the group of 148 operated patients, 95 subtotal thyroidectomies and 64 total thyroidectomies were performed. In 45 patients, the procedure was extended with prophylactic removal of the neck lymph nodes at the same time as total or subtotal thyroidectomy. Reoperation was necessary in 11 children due to the recurrence of the disease or diagnosis of malignant lesions on histopathological examination. In two patients, a biopsy of the second lobe of the thyroid gland was performed due to the abnormality of the lesions found macroscopically. The final histopathological examination showed a benign lesion in the first operated lobe and no pathology in the second lobe in both children (**Figure 1**).

There were 113 (76%) girls with a median age at the time of surgery of 14.95 years. In the boy group, the median age at surgery was 14.86 years. The average time from diagnosis to surgery was 4 months, ranging from 2 weeks to 3 years. The prolonged time from diagnosis to surgery was due to attempts to treat the disease conservatively. Among the operated girls, 80 (70.80%) were euthyroid, 8 (7.80%) presented hyperthyroidism, and 25 (22.12%) were hypothyroid. Among the boys, 26 (74.29%) had euthyroidism, 3 (8.57%) had hyperthyroidism, and 6 (17.14%) had hypothyroidism.

Among 64 children undergoing total thyroidectomy, 26 (40.62%) had indications other than cancer. Detailed indications for thyroid removal and final histopathological results in girls and boys are shown in **Table 2**.

**Table 2:** Comparison of accuracy of initial and final diagnoses in a group of girls and boys.

Gender						
Girls				Boys		
Diagnosis	Initial number of patients	Final number of patients	Concordance factor	Initial number of patients	Final number of patients	Concordance factor
Goiter	76	42	0.87	24	14	0.292
Graves’ disease	3	3	0.0008	0	0	*
Hashimoto’s disease	4	10	0.01	1	3	0.0009
Thyrotoxicosis	3	0	*	1	0	*
Benign thyroid neoplasms, undefined	7	0	0.222^+^	1	0	0.463^+^
Malignant thyroid neoplasms, undefined	25	0	0.66^+^	5	0	0.552^+^
Papillary thyroid carcinoma	5	38	0.14	3	10	0.126
Follicular thyroid carcinoma	1	4	0.05	0	2	*
Medullary thyroid carcinoma	0	2	*	0	0	*
Lymphoepithelial cyst	0	1	*	0	0	*
Sinus histiocytosis	0	2	*	0	0	*
Hyalinizing trabecular adenoma	0	0	*	0	1	*
Burkitt’s lymphoma	0	0	*	0	1	*
Mature teratoma	0	1	*	0	0	*
Thyroid tissue	0	5	*	0	0	*
Thyroid adenoma	0	16	*	0	4	*

*Cannot be counted because there was a value of “0” in the initial/final diagnosis.

^+^When the initial diagnosis was “Malignant thyroid neoplasms, undefined,” and the final diagnosis was a detailed specification of the type of malignant neoplasm, a concordance factor was calculated for the given value. A similar principle was applied for the diagnosis of “Benign thyroid neoplasms, undefined”.

Of the five children with Hashimoto’s goiter, two had symptoms of tracheal compression as an indication for surgery; in another two, the FNAB result indicated the malignant nature of the lesion, and in one, the ultrasound result suggested a malignant process. Of the 100 children with nodular goiter, the indication for thyroidectomy was the cytologic result of FNAB according to the Bethesda classification in 56 patients, which suggested atypia of undetermined significance. In 15 cases, the result indicated suspected follicular neoplasm; in another 19 cases, the huge size of the nodular goiter was the reason for the indication for surgery, and in the remaining 5 children, the worrisome ultrasound image was the indication for surgery. In four patients, thyrotoxicosis was caused by amiodarone treatment.

The initial (preoperative) diagnosis corresponded to the final diagnosis in 68 (42.77%) girls and 22 (13.84%) boys. The malignant lesions in girls on final histopathological examination were diagnosed more often than in the initial diagnosis based on ultrasound imaging and FNAB biopsy (27.43% vs. 38.93%, respectively). A similar situation was also observed in boys (22.86% vs. 37.14%, respectively). Among the 45 neck lymphadenectomies performed, histopathological examination confirmed the involvement of the lymph nodes of the third region in 8 patients and the sixth region in 23 patients. In children with a malignant thyroid lesion, lymph node involvement was demonstrated in 68.9%. In all 54 children with papillary and follicular carcinoma, the stage was defined as I (T1 in 40 patients, T2 in 10 patients, and T3 in 4 patients).

**Figure 2:**
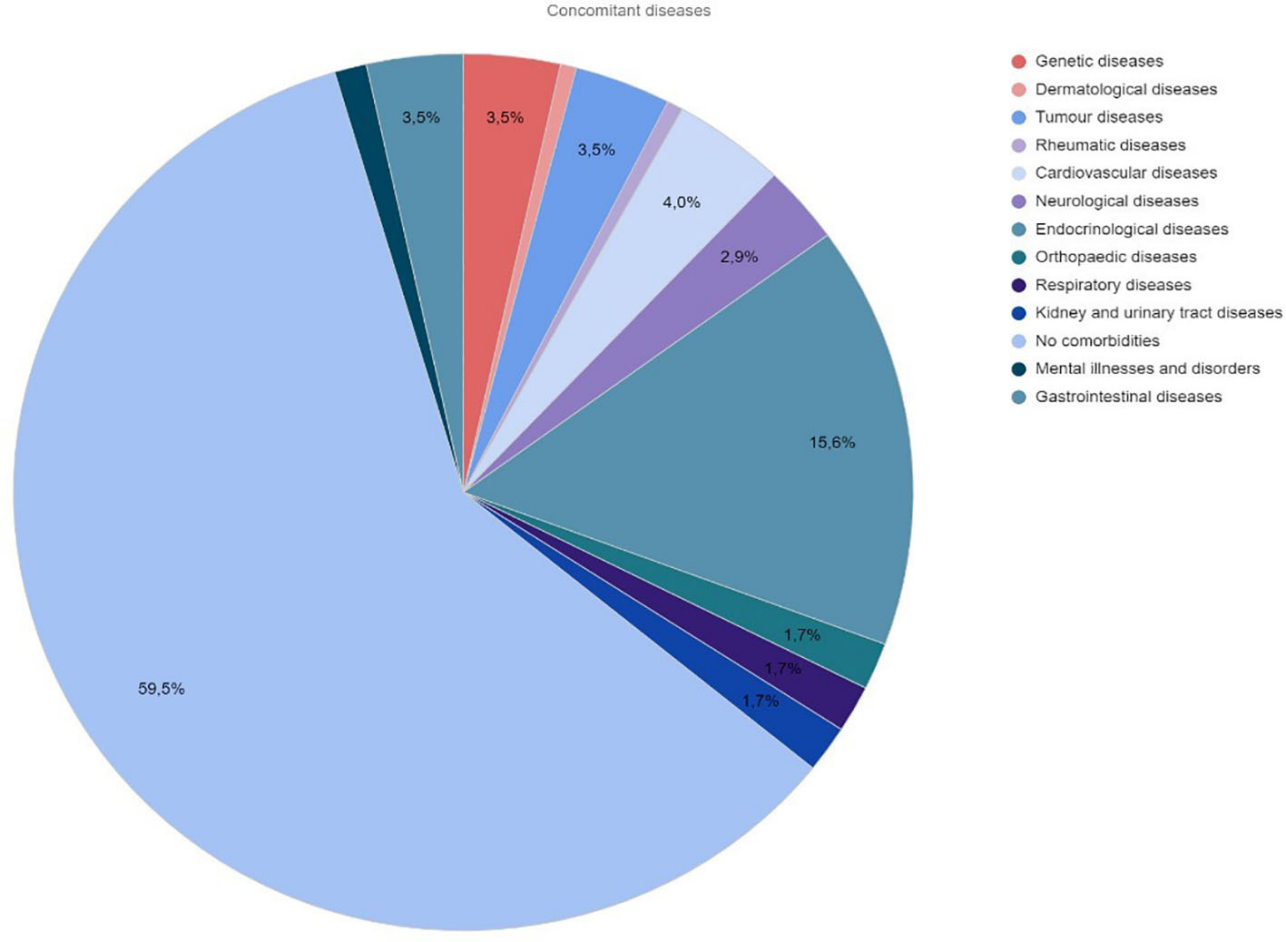
Percentage distribution of comorbidities in children who have undergone thyroidectomy.

Additional health problems were observed in 30.41% of the operated patients. **Figure 2** shows the percentage distribution of comorbidities in the study group of children.

Six children had prior therapy for the oncological disease before thyroid surgery. Because of hyperthyroidism, 11 children required pharmacological preparation for surgery with Lugol’s iodine for 7 days.

The average duration of surgery was 71 min, from 40 to 120 min in the subtotal thyroidectomy group, and 100 min, from 40 to 270 min in the total thyroidectomy group. The prolongation of surgery was related to the advancement of the disease, difficult surgical access, and the lack of experience of the surgical team.

None of the children required blood transfusion during surgery. A blood transfusion was needed on day 4 after surgery in only one child. The patient rated pain on the first postoperative day on a scale of 1 to 10. Most patients rated pain at 1 (107 patients); the maximum value of 5 occurred only in 1 child. The mean value of reported pain was 1.68. Standard pain medications after surgery were paracetamol and metamizole. All children who underwent total thyroidectomy required an intravenous calcium supply for the first 48 h, followed by an oral calcium supply in a dose that depended on the total and ionized calcium level in the body. Two of 54 children diagnosed with malignant neoplasm required subsequent radioiodine therapy. One child diagnosed with Burkitt’s lymphoma required further chemotherapy. Early complications after thyroid surgery were observed in 43 patients (39.05%). Six children had symptoms of transient retrograde laryngeal nerve palsy, which completely resolved in all of them 7–10 days after surgery. Thirty-seven patients developed transient hypoparathyroidism, which resolved completely 2 months after thyroidectomy. Late complications after thyroid surgery were observed in three patients (2.03%). One child developed vocal cord paralysis and required a tracheostomy. Two patients developed hypoparathyroidism and required regular oral calcium supplementation (500 mg three times a day).

**Figure 3:**
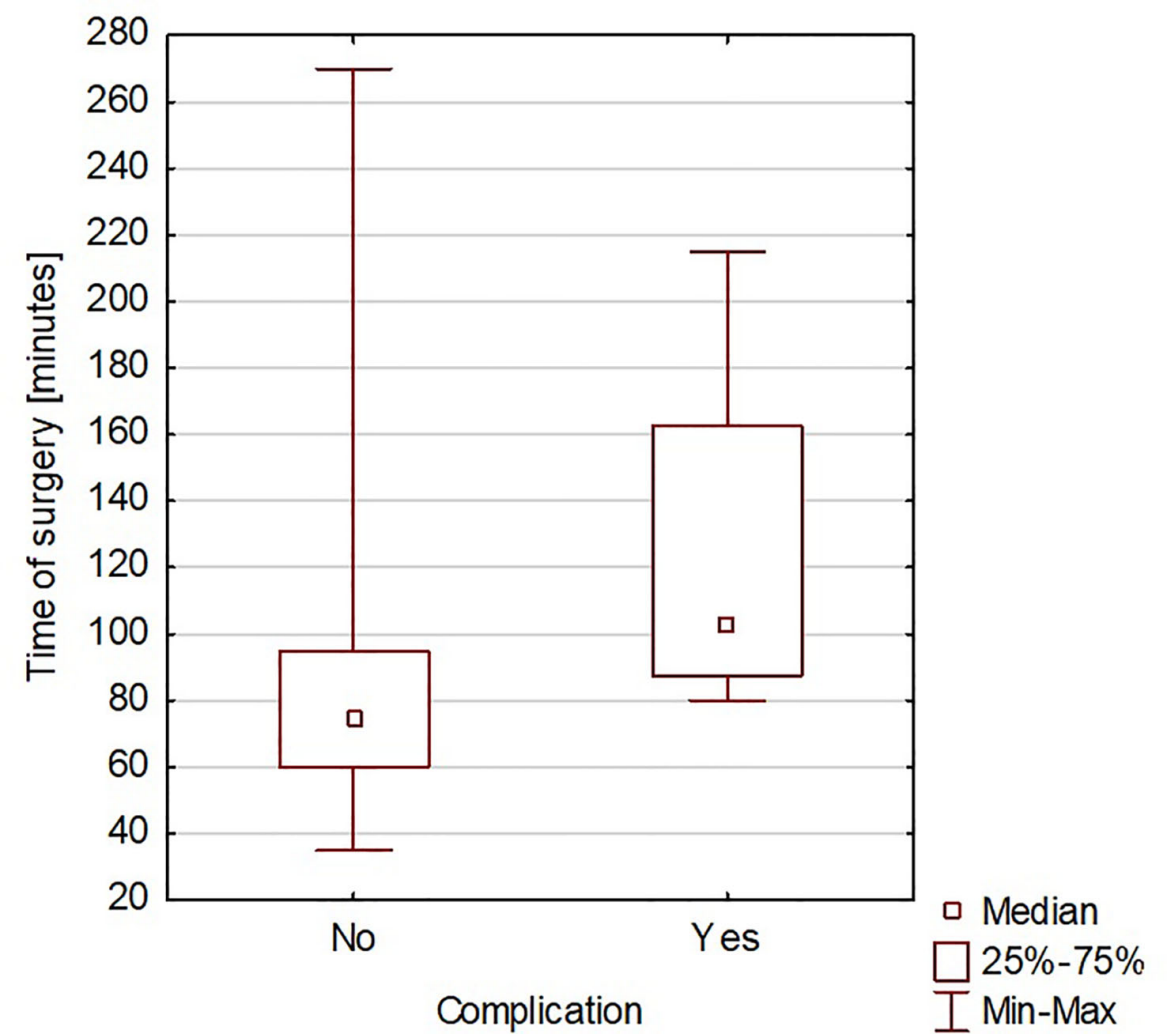
The duration of surgery and the rate of complications.

**Figure 4:**
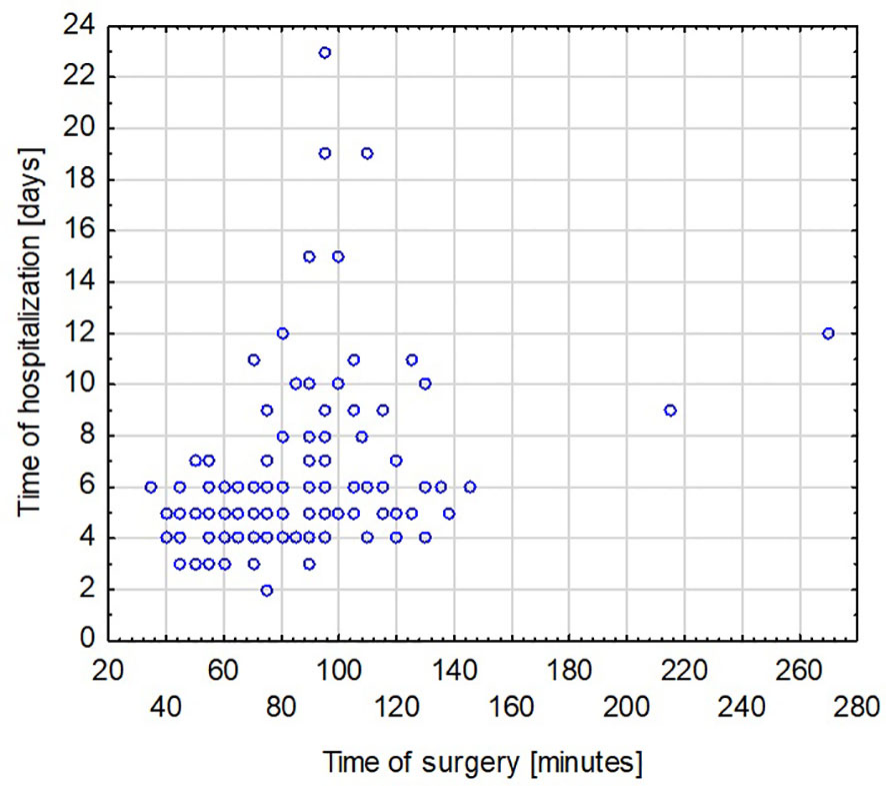
The duration of surgery and the length of hospitalization.

**Figure 5:**
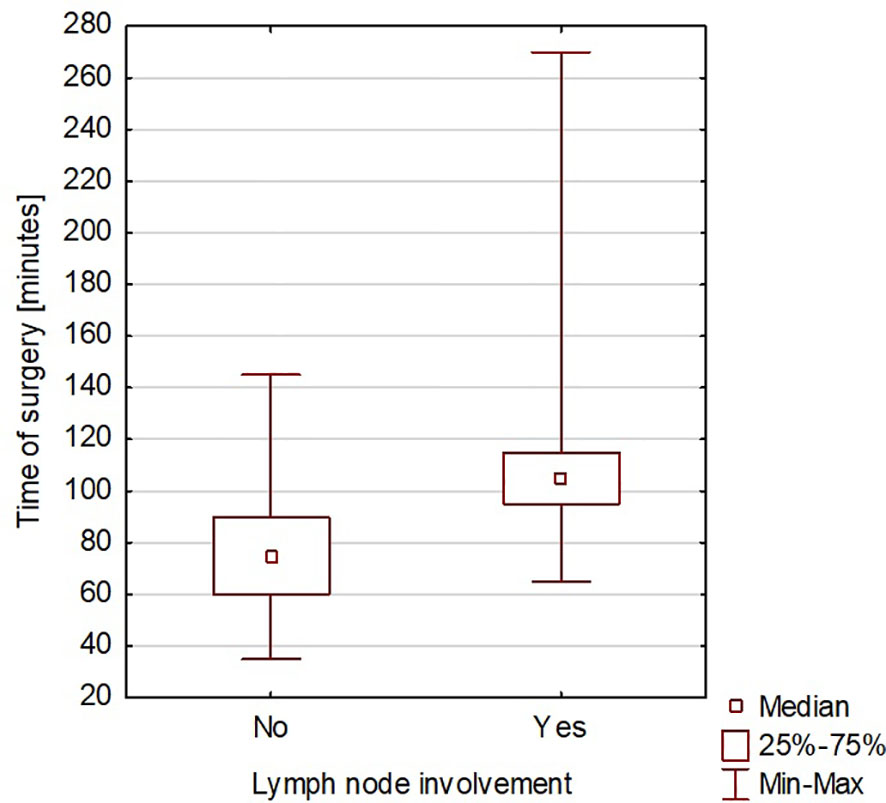
Thyroidectomy with lymph node resection and duration of surgery.

**Figure 6:**
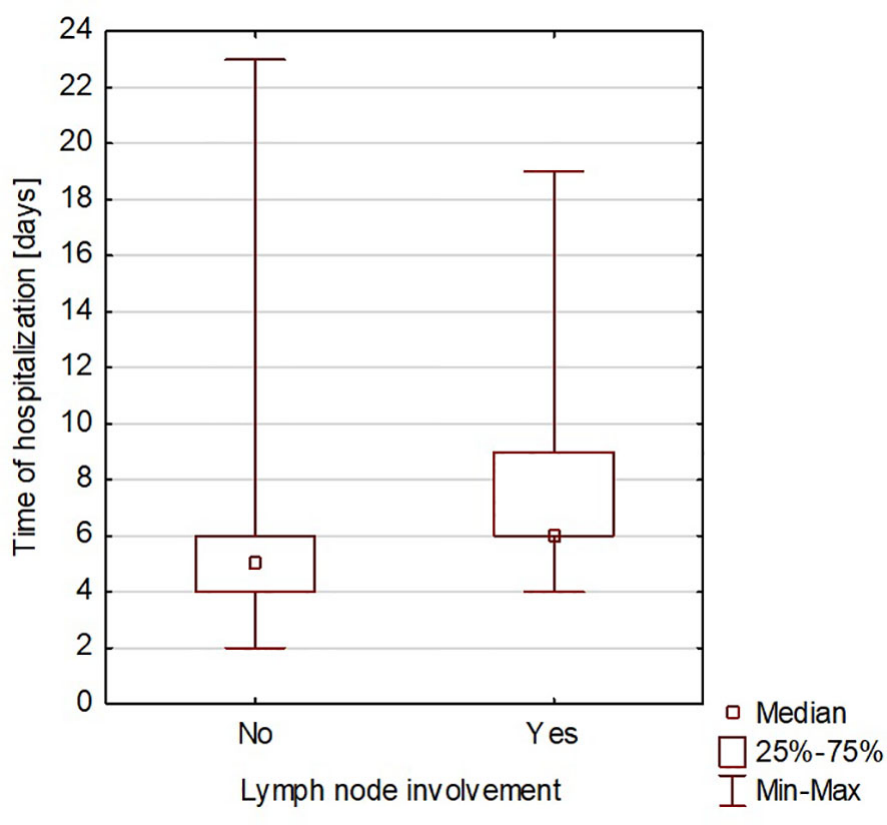
Thyroidectomy with lymph node resection and length of hospitalization.

The duration of hospitalization after surgery ranged from 2 to 23 days, with an average of 6 days. A significant correlation was found between the time of surgery and the rate of complications (Spearman’s rank correlation coefficient = 0.419965) (**Figure 3**). A significant correlation was also found between the duration of surgery and the length of hospitalization (Spearman’s rank correlation coefficient = 0.425831) (**Figure 4**). A significant correlation was also found between thyroidectomy with lymph node resection and duration of surgery, as well as the length of hospitalization (Spearman’s rank correlation coefficient = 0.772595 and 0.675662, respectively) (**Figures 5**, **6**).

## Discussion

Thyroid diseases in children and adolescents are a growing problem that affects an increasing number of patients. Many factors can influence this. Increased awareness and better diagnostics can lead to more appropriate diagnosis and detection of thyroid diseases in children, and increased awareness among doctors and the public about the symptoms and risks of thyroid diseases can result in earlier diagnoses. Increased exposure to various environmental factors, such as air pollution, chemicals, and hormonal substances, can affect thyroid health in children and adults. Dietary changes, such as low intake of iodine, essential for proper thyroid function, can also contribute to thyroid disorders in children. Modern lifestyles, including the pace and level of stress, can affect thyroid health. Stress can affect hormonal regulation, which, in turn, can lead to thyroid disorders (19).

A characteristic feature of thyroid diseases is that they occur mainly in women. The female-to-male ratio is 4–6:1 for autoimmune thyroid diseases and approximately 3–4:1 for thyroid nodules (20). In our group, the number of girls operated on for thyroid diseases was three times higher than for boys. The standard management of thyroid diseases in their early stages is observing the nodules’ growth rate and possible drug treatment (19). Surgery is indicated in selected situations (21). Until qualification for surgery, patients remain under the constant supervision of an endocrinologist. In analyzed patients, the time interval from the diagnosis of thyroid disease to surgical treatment ranged from 2 weeks to 3 years. Operative treatment after such a long time was due to ineffective conservative treatment. An alarming ultrasound image, suggestive of a malignant process, resulted in urgent biopsy and, if the malignant nature of the lesion was confirmed, urgent surgical treatment. Ultrasound parameters such as microcalcifications, hypoechogenicity, lack of halo, increased intratumoral vascularization, nodule shape, or irregular margins have traditionally been associated with an increased risk of malignancy. However, none of these features appear to be reliable enough to diagnose malignancy based on ultrasound alone (22, 23).

It is estimated that approximately 60% of surgeries are for removing nodular goiter, and approximately 20% of procedures are performed for oncological reasons (3, 24). In our material, the most common indications for surgery were goiter (28% of patients) and papillary thyroid carcinoma (26% of children). In 56.61% of all patients, the preoperative diagnosis matched the final histopathological diagnosis. The initial benign recognition based on FNAB may differ from the final diagnosis of the removed thyroid. In the study group, we observed a relatively high malignancy rate of the thyroid gland operated due to multinodular goiter. Similar observations were found by Jenica Su-Ern Yong et al., where the incidence of malignancies in surgically treated multinodular goiter was 14.3% (32 of 223 patients) (25). Such discrepancies between the cytologic result from the biopsy and the histopathological final result from surgery can be explained by the different experiences of the centers in performing FNAB. The children analyzed in the study overwhelmingly had FNAB performed outside our center, which may account for the differences between the initial and final diagnoses. The impact of the centralization of treatment of the patients we operated on the frequency of histopathological results is also worth noting. Our clinic is one of the three higher referral centers for thyroid surgery in children and adolescents; thus, we have many problematic patients. This makes the analyzed group of patients somewhat selective. In the group we studied, the diagnosis of Burkitt’s lymphoma and teratoma in the operated thyroid was surprising.

Current surgical techniques include classic approaches such as subtotal or total thyroidectomy with or without lymph node removal. Videoscopic and endoscopic procedures are also available (26–28). These two methods are not used in our center. The available literature rarely describes them in children (29).

As described in the literature, complications associated with retrobulbar nerve injury have decreased due to the greater availability of intraoperative neuromonitoring (30). In our center, every thyroid surgery is performed with neuromonitoring as standard. According to Motos-Micó et al., the availability of neuromonitoring reduced the number of described complications in his study group to zero (31). In our group, transient symptoms of laryngeal nerve palsy occurred in six children (4% of patients) despite using neuromonitoring. Unilateral vocal cord paralysis manifested by dysphonia, dysphonia, and difficulty swallowing was observed in only one child. The other most frequently observed complication was transient hypoparathyroidism, which occurred in 37% of children. Compared to the literature data, these are comparable in the incidence of retrobulbar laryngeal nerve injury and hypoparathyroidism in the pediatric group, depending on the center performing the study (32, 33). Willobee et al. showed that the most common complications were hypocalcemia and laryngeal nerve injury, with rates of 9% and 3%, respectively (3). Hanba et al. described that vocal cord paralysis was found in 1.7% of children after thyroidectomy. Postoperative hypocalcemia occurred in almost 20% of children undergoing total thyroidectomy (33).

The correlation we observed between the duration of surgery and the presence of intra- or postoperative complications is worth noting. This can be explained by the more difficult course of the surgical procedure. Hanba et al. reported that children under 1 year of age stayed in the hospital significantly longer, and patients aged 1 to 5 years stayed much longer than those aged 6 to 20 years (33). In our study, we did not observe a similar relationship. Willobee et al. showed that compared with partial thyroidectomy, total thyroidectomy was associated with an increased rate of postoperative complications. Moreover, the median length of hospital stay was significantly higher in patients after total thyroidectomy (32). In our observations, prolonged surgery time was also associated with more extended hospital stays. This was also confirmed in the study by Willobee et al. (32). We showed a correlation between lymphadenectomy and more extensive surgery with longer hospitalization time. Martucci et al. presented that according to international guidelines, prophylactic central and lateral neck excision is not recommended (34). They stated that it should only be performed in patients with malignant cytology (grade 4, 5, or 6 on the Bethesda scale) or when there is clear clinical evidence of extrathyroidal node involvement or locoregional metastasis based on preoperative staging or intraoperative findings. In the study by Martucia et al., 153 children (60%) underwent lymphadenectomy, and 85.6% had lymph node metastases (34). In the material we analyzed, 45 patients (30.41% of all patients) underwent lymphadenectomy, and metastasis was confirmed in 31 children (68.9%).

Thyroid surgeries in our center are performed by teams led by three experienced surgeons. The experience of the operating team and its impact on the number of complications during thyroidectomy are widely emphasized in the available literature (35, 36).

## Conclusion

Cooperation in a multidisciplinary team ensures optimal treatment results for patients. With special care, removal of the thyroid gland is a safe procedure with few complications, but the surgeon’s experience in performing thyroid surgery in children is crucial. Cytologic result and final histopathological verification, especially in the case of the slightest suspicion of thyroid cancer, are crucial. Qualification for surgery should adhere to the Bethesda classification. For thyroid conditions warranting surgical intervention, a follow-up fine-needle biopsy with cytologic evaluation should be conducted before reaching a final decision on thyroidectomy. As per the Bethesda classification, a cytologic reassessment should be carried out at a specialized referral center for thyroidectomy.

## Data availability statement

The raw data supporting the conclusions of this article will be made available by the authors, without undue reservation.

## Ethics statement

The studies involving humans were approved by the Bioethics Committee of the Poznan University of Medical Sciences (Resolution no. KB-752/23, 22 September 2023). The studies were conducted in accordance with the local legislation and institutional requirements. Written informed consent for participation in this study was provided by the participants’ legal guardians/next of kin.

## Author contributions

PS-S: Conceptualization, Data curation, Formal analysis, Investigation, Methodology, Project administration, Software, Visualization, Writing – original draft. DD: Data curation, Methodology, Software, Visualization, Writing – original draft, Formal analysis, Funding acquisition. PR: Data curation, Investigation, Software, Writing – original draft, Funding acquisition. IA: Data curation, Methodology, Software, Writing – original draft, Funding acquisition, Investigation. MR: Data curation, Methodology, Software, Writing – original draft, Funding acquisition, Investigation. DJ-L: Conceptualization, Formal analysis, Funding acquisition, Methodology, Supervision, Writing – review & editing. MN: Conceptualization, Funding acquisition, Investigation, Supervision, Writing – review & editing. JH: Data curation, Funding acquisition, Investigation, Supervision, Writing – review & editing. PM: Conceptualization, Formal analysis, Funding acquisition, Investigation, Supervision, Writing – review & editing.
